# Supplementary figures and images for: Histopathological change of age-related hearing loss in female advance-aged CBA/CaJ mice
Source: PLoS One. 2025 Oct 7;20(10):e0334021. doi: 10.1371/journal.pone.0334021 (PMC12503338; doi:10.1371/journal.pone.0334021)

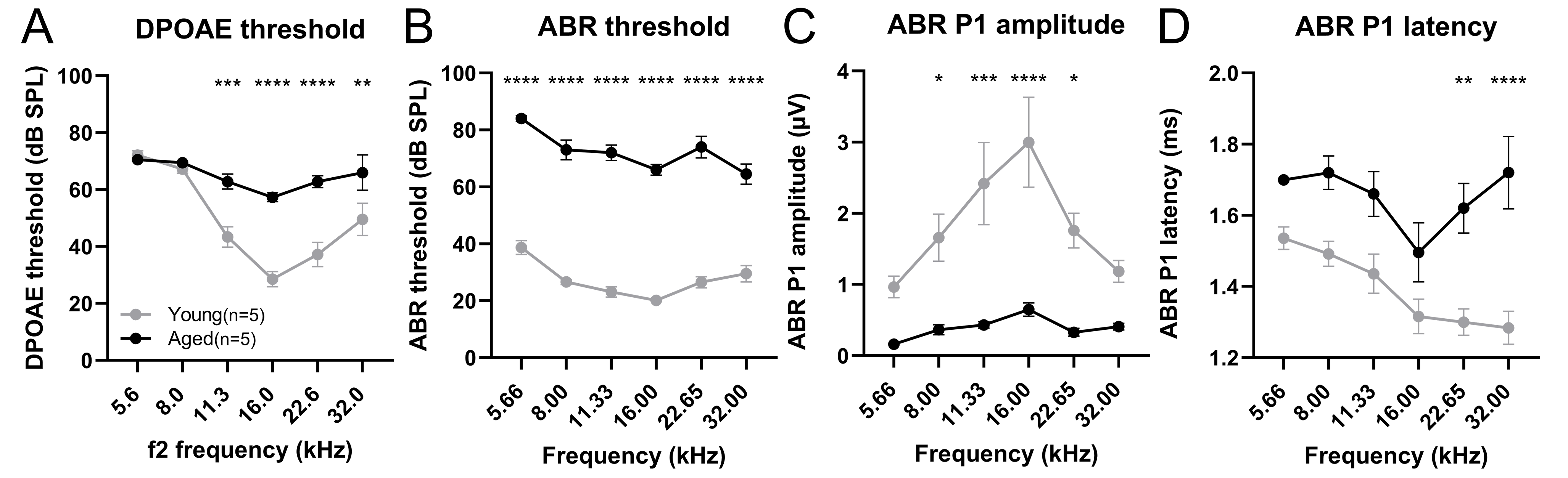

Supplement: S1 Fig — (A) DPOAE thresholds. (B) ABR thresholds. (C) ABR P1 amplitude. (D) ABR P1 latency. The number of subjects is 5 mice per group. The overall results are consistent with per-ear comparing analysis. Error bars represent standard error of mean. Asterisks indicate significant differences. *p < 0.05, **p < 0.01, ***p < 0.001, ****p < 0.0001. (TIF) [file pone.0334021.s001.tif]

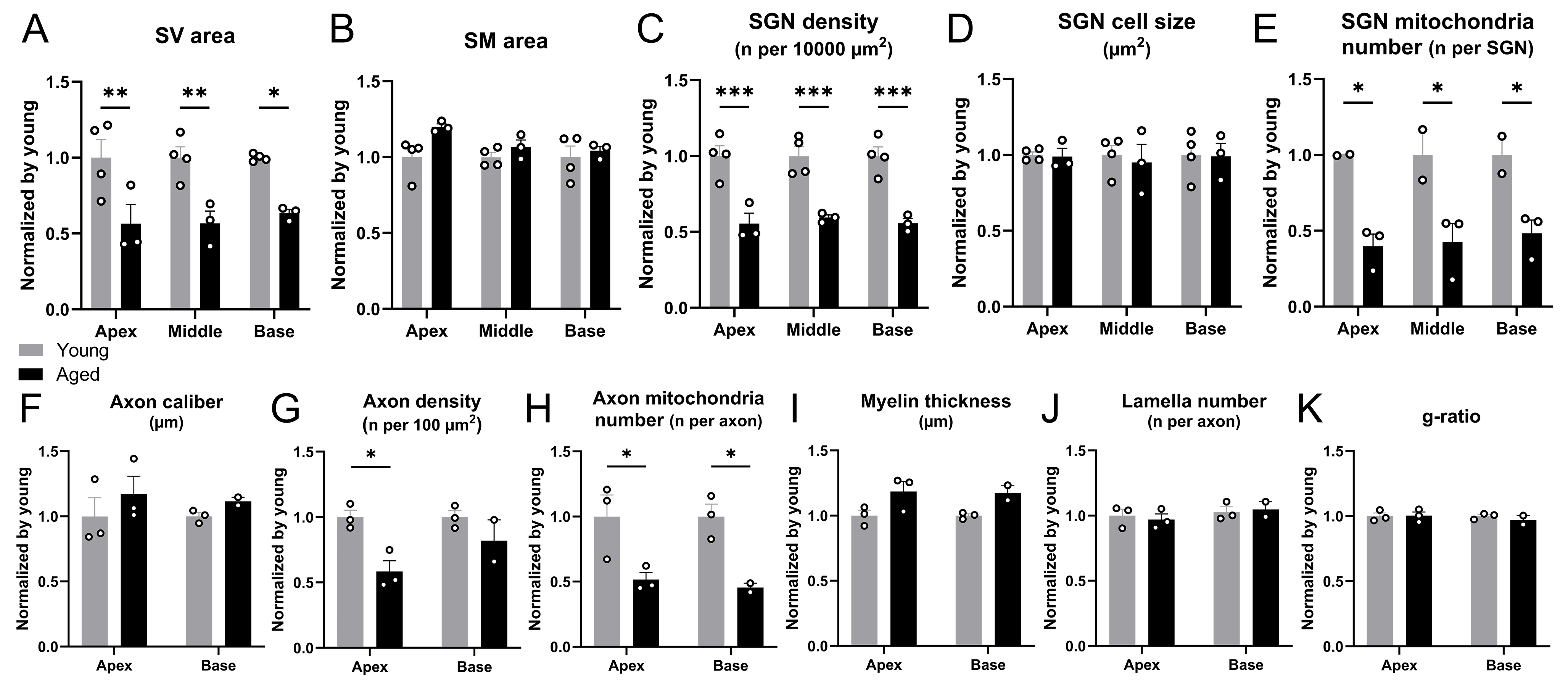

Supplement: S3 Fig — (A) SV area. (B) SM area. (C) SGN density. (D) SGN cell size. (E) SGN mitochondria number. (F) Axon caliber. (G) Axon density. (H) Axon mitochondria number. (I) Myelin thickness. (J) Lamella number. (K) g-ratio. The number of subjects is listed in S4 Table. The overall results are consistent with per-ear comparing analysis. Error bars represent standard error of mean. Asterisks indicate significant differences. *p < 0.05, **p < 0.01, ***p < 0.001. (TIF) [file pone.0334021.s003.tif]
