# Supplementary material for: Histopathological change of age-related hearing loss in female advance-aged CBA/CaJ mice
Source: PLoS One. 2025 Oct 7;20(10):e0334021. doi: 10.1371/journal.pone.0334021 (PMC12503338; doi:10.1371/journal.pone.0334021)
Supplement: S2 Table — (DOCX) [file pone.0334021.s002.docx]

DPOAE threshold (Young: N = 5 mice, Aged: N = 5 mice)

| Two-way ANOVA  (frequency x age) | | Interaction  F (DFn, DFd) | *p* value |  |
| --- | --- | --- | --- | --- |
|  |  | F (5.48) = 7.18 | <0.0001**** |  |
| Šidák post hoc test  kHz | Young  Mean ± SEM | Aged  Mean ± SEM | *p* value | Cohen's d |
| 5.6 | 72.12 ± 0.39 | 70.58 ± 0.43 | 0.9997 | 0.62 |
| 8.0 | 67.17 ± 0.38 | 69.46 ± 0.038 | 0.997 | 1.02 |
| 11.3 | 43.39 ± 1.21 | 62.83 ± 2.60 | 0.0006*** | 2.78 |
| 16.0 | 28.52 ± 1.13 | 57.31 ± 1.52 | <0.0001**** | 5.90 |
| 22.6 | 37.20 ± 1.57 | 62.78 ± 2.06 | <0.0001**** | 3.41 |
| 32.0 | 49.55 ± 1.80 | 65.99 ± 6.24 | 0.0049** | 1.23 |

ABR threshold (Young: N = 5 mice, Aged: N = 5 mice)

| Two-way ANOVA  (frequency x age) | | Interaction  F (DFn, DFd) | *p* value |  |
| --- | --- | --- | --- | --- |
|  |  | F (5.48) = 2.06 | 0.087 |  |
| Šidák post hoc test  kHz | Young  Mean ± SEM | Aged  Mean ± SEM | *p* value | Cohen's d |
| 5.6 | 38.70 ± 0.86 | 84.00 ± 1.00 | <0.0001**** | 11.01 |
| 8.0 | 26.60 ± 0.37 | 73.00 ± 3.48 | <0.0001**** | 8.18 |
| 11.3 | 23.10 ± 0.84 | 72.00 ± 2.67 | <0.0001**** | 9.61 |
| 16.0 | 20.10 ± 0.050 | 66.00 ± 1.87 | <0.0001**** | 15.49 |
| 22.6 | 26.50 ± 0.84 | 74.00 ± 3.76 | <0.0001**** | 7.11 |
| 32.0 | 29.50 ± 1.18 | 64.50 ± 3.57 | <0.0001**** | 4.83 |

ABR P1 amplitude (Young: N = 5 mice, Aged: N = 5 mice)

| Two-way ANOVA  (frequency x age) | | Interaction  F (DFn, DFd) | *p* value |  |
| --- | --- | --- | --- | --- |
|  |  | F (5.42) = 1.84 | 0.13 |  |
| Šidák post hoc test  kHz | Young  Mean ± SEM | Aged  Mean ± SEM | *p* value | Cohen's d |
| 5.6 | 0.97 ± 0.34 | 0.16 | 0.86 | NA |
| 8.0 | 1.66 ± 0.57 | 0.37 ± 0.071 | 0.040* | 2.28 |
| 11.3 | 2.42 ± 0.83 | 0.43 ± 0.044 | 0.0002*** | 2.17 |
| 16.0 | 3.00 ± 0.81 | 0.65 ± 0.093 | <0.0001**** | 2.33 |
| 22.6 | 1.76 ± 0.41 | 0.33 ± 0.055 | 0.018* | 3.43 |
| 32.0 | 1.19 ± 0.32 | 0.41 ± 0.048 | 0.38 | 3.03 |

ABR P1 latency (Young: N = 5 mice, Aged: N = 5 mice)

| Two-way ANOVA  (frequency x age) | | Interaction  F (DFn, DFd) | *p* value |  |
| --- | --- | --- | --- | --- |
|  |  | F (5.42) = 1.31 | 0.28 |  |
| Šidák post hoc test  kHz | Young  Mean ± SEM | Aged  Mean ± SEM | *p* value | Cohen's d |
| 5.6 | 1.54 ± 0.058 | 1.70 | 0.84 | NA |
| 8.0 | 1.49 ± 0.064 | 1.72 ± 0.047 | 0.078 | 2.67 |
| 11.3 | 1.44 ± 0.10 | 1.66 ± 0.063 | 0.060 | 1.69 |
| 16.0 | 1.32 ± 0.094 | 1.50 ± 0.083 | 0.20 | 1.19 |
| 22.6 | 1.30 ± 0.072 | 1.62 ± 0.070 | 0.0047** | 2.89 |
| 32.0 | 1.28 ± 0.091 | 1.72 ± 0.10 | <0.0001**** | 2.47 |

Note: The number included in the ABR P1 analysis was reduced at several frequencies in the aged group because no ABR responses were observed, even at the highest stimulus level (80dB SPL). The exact number of included ABR P1 comparing analysis was as follows:

5.6 kHz: N = 1 mice, 8.0 kHz: N = 4 mice, 22.65 kHz: N = 4 mice
