# Supplementary material for: Histopathological change of age-related hearing loss in female advance-aged CBA/CaJ mice
Source: PLoS One. 2025 Oct 7;20(10):e0334021. doi: 10.1371/journal.pone.0334021 (PMC12503338; doi:10.1371/journal.pone.0334021)
Supplement: S4 Table — (DOCX) [file pone.0334021.s004.docx]

SV area normalized by young (Young: N = 4 mice, Aged: N = 3 mice)

| Two-way ANOVA  (cochlear turn x age) | | Interaction  F (DFn, DFd) | *p* value |  |
| --- | --- | --- | --- | --- |
|  |  | F (2, 15) = 0.10 | 0.90 |  |
| Šidák post hoc test  Cochlear turn | Young  Mean ± SEM | Aged  Mean ± SEM | *p* value | Cohen's d |
| Apex | 1.00 ± 0.24 | 0.56 ± 0.13 | 0.0080** | 1.87 |
| Middle | 1.00 ± 0.14 | 0.57 ± 0.081 | 0.0083** | 3.03 |
| Base | 1.00 ± 0.024 | 0.63 ± 0.026 | 0.025* | 10.78 |

SM area normalized by young (Young: N = 4 mice, Aged: N = 3 mice)

| Two-way ANOVA  (cochlear turn x age) | | Interaction  F (DFn, DFd) | *p* value |  |
| --- | --- | --- | --- | --- |
|  |  | F (2, 15) = 1.27 | 0.31 |  |
| Šidák post hoc test  Cochlear turn | Young  Mean ± SEM | Aged  Mean ± SEM | *p* value | Cohen's d |
| Apex | 1.00 ± 0.13 | 1.20 ± 0.020 | 0.052 | 1.97 |
| Middle | 1.00 ± 0.060 | 1.07 ± 0.046 | 0.77 | 0.96 |
| Base | 1.00 ± 0.15 | 1.04 ± 0.029 | 0.92 | 0.37 |

SGN density normalized by young (Young: N = 4 mice, Aged: N = 3 mice)

| Two-way ANOVA  (cochlear turn x age) | | Interaction  F (DFn, DFd) | *p* value |  |
| --- | --- | --- | --- | --- |
|  |  | F (2, 15) = 0.069 | 0.93 |  |
| Šidák post hoc test  Cochlear turn | Young  Mean ± SEM | Aged  Mean ± SEM | *p* value | Cohen's d |
| Apex | 1.00 ± 0.14 | 0.55 ± 0.070 | 0.0003*** | 3.42 |
| Middle | 1.00 ± 0.13 | 0.59 ± 0.018 | 0.0007*** | 4.04 |
| Base | 1.00 ± 0.12 | 0.56 ± 0.031 | 0.0003*** | 4.47 |

SGN cell size normalized by young (Young: N = 4 mice, Aged: N = 3 mice)

| Two-way ANOVA  (cochlear turn x age) | | Interaction  F (DFn, DFd) | *p* value |  |
| --- | --- | --- | --- | --- |
|  |  | F (2, 15) = 0.051 | 0.95 |  |
| Šidák post hoc test  Cochlear turn | Young  Mean ± SEM | Aged  Mean ± SEM | *p* value | Cohen's d |
| Apex | 1.00 ± 0.039 | 0.99 ± 0.054 | 0.9995 | 0.16 |
| Middle | 1.00 ± 0.12 | 0.95 ± 0.12 | 0.95 | 0.31 |
| Base | 1.00 ± 0.16 | 0.99 ± 0.085 | 0.9997 | 0.059 |

SGN mitochondria number normalized by young (Young: N = 2 mice, Aged: N = 3 mice)

| Two-way ANOVA  (cochlear turn x age) | | Interaction  F (DFn, DFd) | *p* value |  |
| --- | --- | --- | --- | --- |
|  |  | F (2, 9) = 0.080 | 0.92 |  |
| Šidák post hoc test  Cochlear turn | Young  Mean ± SEM | Aged  Mean ± SEM | *p* value | Cohen's d |
| Apex | 1.00 ± 0.039 | 1.00 ± 0.081 | 0.40±0.081 | 0.011 |
| Middle | 1.00 ± 0.12 | 1.00 ± 0.12 | 0.42±0.24 | 0.015 |
| Base | 1.00 ± 0.16 | 1.00 ± 0.086 | 0.48±0.17 | 0.027 |

Axon density normalized by young (Young: N = 2 mice, Aged: N = 3 (apex) / 2 (base) mice)

| Two-way ANOVA  (cochlear turn x age) | | Interaction  F (DFn, DFd) | *p* value |  |
| --- | --- | --- | --- | --- |
|  |  | F (1, 7) = 2.090 | 0.19 |  |
| Šidák post hoc test  Cochlear turn | Young  Mean ± SEM | Aged  Mean ± SEM | *p* value | Cohen's d |
| Apex | 1.00 ± 0.093 | 0.58 ± 0.083 | 0.013* | 3.43 |
| Base | 1.00 ± 0.085 | 0.82 ± 0.16 | 0.33 | 1.22 |

Axon caliber normalized by young (Young: N = 2 mice, Aged: N = 3 (apex) / 2 (base) mice)

| Two-way ANOVA  (cochlear turn x age) | | Interaction  F (DFn, DFd) | *p* value |  |
| --- | --- | --- | --- | --- |
|  |  | F (1, 7) = 0.062 | 0.81 |  |
| Šidák post hoc test  Cochlear turn | Young  Mean ± SEM | Aged  Mean ± SEM | *p* value | Cohen's d |
| Apex | 1.00 ± 0.25 | 1.17 ± 0.14 | 0.50 | 0.71 |
| Base | 1.00 ± 0.050 | 1.12 ± 0.031 | 0.77 | 2.42 |

Axon mitochondria number normalized by young (Young: N = 2 mice, Aged: N = 3 (apex) / 2 (base) mice)

| Two-way ANOVA  (cochlear turn x age) | | Interaction  F (DFn, DFd) | *p* value |  |
| --- | --- | --- | --- | --- |
|  |  | F (1, 7) = 0.073 | 0.79 |  |
| Šidák post hoc test  Cochlear turn | Young  Mean ± SEM | Aged  Mean ± SEM | *p* value | Cohen's d |
| Apex | 1.00 ± 0.29 | 0.52 ± 0.054 | 0.030* | 2.26 |
| Base | 1.00 ± 0.17 | 0.45 ± 0.034 | 0.029* | 3.95 |

Myelin thickness normalized by young (Young: N = 2 mice, Aged: N = 3 (apex) / 2 (base) mice)

| Two-way ANOVA  (cochlear turn x age) | | Interaction  F (DFn, DFd) | *p* value |  |
| --- | --- | --- | --- | --- |
|  |  | F (1, 7) = 0.0087 | 0.93 |  |
| Šidák post hoc test  Cochlear turn | Young  Mean ± SEM | Aged  Mean ± SEM | *p* value | Cohen's d |
| Apex | 1.00 ± 0.072 | 1.19 ± 0.077 | 0.070 | 1.73 |
| Base | 1.00 ± 0.025 | 1.18 ± 0.057 | 0.12 | 3.43 |

Myelin lamella number normalized by young (Young: N = 2 mice, Aged: N = 3 (apex) / 2 (base) mice)

| Two-way ANOVA  (cochlear turn x group) | | Interaction  F (DFn, DFd) | *p* value |  |
| --- | --- | --- | --- | --- |
|  |  | F (1, 7) = 0.68 | 0.44 |  |
| Šidák post hoc test  Cochlear turn | Young  Mean ± SEM | Aged  Mean ± SEM | *p* value | Cohen's d |
| Apex | 1.00 ± 0.085 | 0.97 ± 0.042 | 0.88 | 0.37 |
| Base | 1.00 ± 0.070 | 1.05 ± 0.058 | 0.76 | 0.65 |

g-ratio normalized by young (Young: N = 2 mice, Aged: N = 3 (apex) / 2 (base) mice)

| Two-way ANOVA  (cochlear turn x group) | | Interaction  F (DFn, DFd) | *p* value |  |
| --- | --- | --- | --- | --- |
|  |  | F (1, 7) = 0.47 | 0.52 |  |
| Šidák post hoc test  Cochlear turn | Young  Mean ± SEM | Aged  Mean ± SEM | *p* value | Cohen's d |
| Apex | 1.00 ± 0.041 | 1.00 ± 0.028 | 0.99 | 0.085 |
| Base | 1.00 ± 0.023 | 0.97 ± 0.034 | 0.69 | 0.90 |
