# Supplementary material for: Histopathological change of age-related hearing loss in female advance-aged CBA/CaJ mice
Source: PLoS One. 2025 Oct 7;20(10):e0334021. doi: 10.1371/journal.pone.0334021 (PMC12503338; doi:10.1371/journal.pone.0334021)
Supplement: Attachment — Submitted filename: Response to Reviewers.docx [file pone.0334021.s013.docx]

# Response to Reviewers

Dr. Miriam A. Hickey
Academic Editor, PLOS ONE

Re: Manuscript ID PONE-S-25-27786
Title: Histopathological change of age-related hearing loss in advance-aged CBA/CaJ mice

Dear Dr. Hickey,

Thank you very much for your careful review and the helpful feedback provided during the initial check. We have carefully revised the manuscript in accordance with your suggestions and are submitting the updated version for your further consideration. Please find below our point-by-point responses to the issues raised.

## Journal Requirements:

1. Formatting:

“Please ensure that your manuscript meets PLOS ONE's style requirements, including those for file naming.”

We apologize for not using the correct formatting in our original submission. The manuscript has been reformatted entirely to follow the PLOS ONE style requirements.

2. Animal Experimentation Details:

“To comply with PLOS ONE submissions requirements, in your Methods section, please provide additional information regarding the experiments involving animals and ensure you have included details on (1) methods of sacrifice, and (2) efforts to alleviate suffering.”

In accordance with your guidance, we have revised the Materials and Methods section to include (1) the method of euthanasia and (2) efforts made to minimize animal suffering. Thank you for bringing this to our attention.

3. Funding Disclosure:

“Please state what role the funders took in the study. If the funders had no role, please state: "The funders had no role in study design, data collection and analysis, decision to publish, or preparation of the manuscript.”

As the funders had no role in the study design, data collection and analysis, decision to publish, or preparation of the manuscript, we have added the following statement to the cover letter:
“The funders had no role in study design, data collection and analysis, decision to publish, or preparation of the manuscript.”

## Editor’s Comment on Sex Matching in Experimental Animals:

“The old group of mice appears to comprise nine female CBA/CaJ mice 104 aged between 129 and 138 weeks whereas the young group appears to comprise seven male CBA/CaJ mice aged 9-14 weeks. Due to the established sex-based differences in hearing and hearing loss in males versus females (humans and mice), the authors are asked to provide a sex-matched young group, for comparisons.”

We sincerely apologize for the incorrect description regarding the sex of the animals used in our study. The original manuscript incorrectly stated that the young group consisted of male mice, while the aged group consisted of females. In fact, both groups were composed of female CBA/CaJ mice. This inconsistency was a serious oversight on our part. We have corrected the manuscript accordingly. We greatly appreciate your careful review and the opportunity to rectify this error.

Please do not hesitate to contact us if any further clarification is needed. We thank you again for your attention and support of our work.

Sincerely,
Kunio Mizutari, M.D., Ph.D.
On behalf of all authors
