# Supplementary material for: Histopathological change of age-related hearing loss in female advance-aged CBA/CaJ mice
Source: PLoS One. 2025 Oct 7;20(10):e0334021. doi: 10.1371/journal.pone.0334021 (PMC12503338; doi:10.1371/journal.pone.0334021)
Supplement: Attachment — Submitted filename: renamed_30fcc.docx [file pone.0334021.s014.docx]

The author of this article studied the changes in inner ear structure and auditory function in an animal model of age-related hearing loss. Although this study is not a intervention study and the innovation was limited, the research content has certain value. I have some questions as follows.

1. Please mark the references in the first line of page 4 of the manuscript.
2. Please use signs such as arrows to indicate the relevant and explanatory information described in the legend text of Figures 3A and 7B.
3. The statement in the line 477-479 is incomprehensible. Please elaborate on this part in detail.
