# Supplementary material for: Histopathological change of age-related hearing loss in female advance-aged CBA/CaJ mice
Source: PLoS One. 2025 Oct 7;20(10):e0334021. doi: 10.1371/journal.pone.0334021 (PMC12503338; doi:10.1371/journal.pone.0334021)
Supplement: Attachment — Submitted filename: Response_to_Reviewers.docx [file pone.0334021.s015.docx]

**Point to Point Response to the Reviewers**

Manuscript Number: PONE-D-25-21282R1

We would like to thank the editor and the reviewers for their comments and believe they have helped to strengthen the manuscript. We have made edits and improved our manuscript as suggested. In the following, we provide a point-to-point response to every comment raised. We have highlighted portions of the manuscript that have been updated in response to the reviewers’ comments.

Reviewer #1: 
Major concerns
1. I believe this is a study comparing young and super-aged CBA/CaJ mice. However, if middle-aged mice were also included, it might have been possible to determine where age-related changes begin within the cochlea. Please add this point to the limitations section.
Response: Thank you for this comment. We agree with you completely that using middle-aged mice would have provided a more comprehensive understanding of the progression of age-related cochlear degeneration. A previous study by Sergeyenko et al. investigated the auditory function and cochlear histological changes, such as HCs, synapses, and SGNs, across multiple age groups in CBA/CaJ mice^1^. Our study has evaluated additional aspects of cochlear pathology, including the stria vascularis, cochlear hydrops, myelination of ANFs, and mitochondrial morphology, with the aim of comprehensively assessing advanced-aged mice. However, as the reviewer correctly pointed out, the lack of a middle-aged group limited the ability of our study to determine the precise onset and progression of these degenerative changes. We have added this description to the limitations section. (P.38, L.680-685)

*Reference #1: Sergeyenko Y, Lall K, Liberman MC, Kujawa SG. Age-related cochlear synaptopathy: an early-onset contributor to auditory functional decline. J Neurosci. 2013; 33(34): 13686-13694. https://doi.org/10.1523/JNEUROSCI.1783-13.2013. PMID: 23966690.*

2. While it has been pointed out that mitochondrial function and metabolism within the cochlea are important, the discussion of systemic oxidative stress alone may weaken the argument.　There are several papers that have measured actual cochlear metabolism, so please cite them and add them to the discussion.
Response: We thank the reviewer for the insightful and constructive comment. We fully agree that we should consider actual cochlear metabolism in addition to systemic oxidative stress. A previous study reported that mitochondrial aminoacyl-tRNA synthetase-KO mice showed elevated reactive oxygen species levels, activation of the mitochondrial apoptotic pathway, and morphologically abnormal mitochondria in cochlear hair cells^2^. Another study demonstrated that the enzyme catalyzing the deposition of 2-methylthio modifications in mt-tRNA KO mice showed a progressive accumulation of oxidative stress related to HC loss, SGN loss, and mitochondrial morphological changes^3^. These histological deformations were also observed in the current study, including a reduction in the mitochondrial number in the SGN soma and axons, as well as mitochondrial malformations, such as ballooning and damaged cristae. Therefore, we considered that a potentially high level of oxidative stress exists in the cochlea along with a systemically high level of ROS. We have added these citations and descriptions in the Discussion section. (P.37, L.661-667)

*Reference #2: Xu P, Wang L, Peng H, Liu H, Liu H, Yuan Q, et al. Disruption of Hars2 in Cochlear Hair Cells Causes Progressive Mitochondrial Dysfunction and Hearing Loss in Mice. Front Cell Neurosci. 2021; 15: 804345. https://doi.org/10.3389/fncel.2021.804345. PMID: 34975414.*

*Reference #3: Miwa T, Wei F-y, Tomizawa K. Cdk5 regulatory subunit-associated protein 1 knockout mice show hearing loss phenotypically similar to age-related hearing loss. Molecular Brain. 2021; 14(1). https://doi.org/10.1186/s13041-021-00791-w.*

3. Authors concludes that long-term acoustic exposure is a cause of age-related hearing loss, but what level of acoustic exposure is considered acceptable? Would hearing be better preserved without acoustic exposure? Please discuss previous reports on long-term acoustic exposure.
Response: We would like to thank the reviewer for this important comment and agree that identifying an acceptable level of long-term acoustic exposure is the key to understanding and preventing age-related hearing loss. Although few studies have directly explored safe ambient sound levels for long-term exposure, several studies have provided valuable insights. Tarnowki et al. showed that gerbils raised in a quiet environment (<41 dBA) retained IHCs and OHCs that were comparable to those of young group^4^. Additionally, Rosen et al.. observed that individuals in an isolated primitive area with ambient sound levels below 40 dB maintained better hearing thresholds in old age than those in industrialized settings^5^. These findings therefore suggest that chronic exposure to above ~40 dB SPL may contribute to age-related hearing loss, whereas lower levels may help preserve auditory function. We have added this information in the revised manuscript. (P.32, L.578-585)

*Reference #4: Tarnowski BI, Schmiedt RA, Hellstrom LI, Lee FS, Adams JC. Age-related changes in cochleas of mongolian gerbils. Hear Res. 1991; 54(1): 123-134. https://doi.org/10.1016/0378-5955(91)90142-v. PMID: 1917712.*

*Reference #5: Rosen S, Bergman M, Plester D, El-Mofty A, Satti MH. Presbycusis study of a relatively noise-free population in the Sudan. Ann Otol Rhinol Laryngol. 1962; 71: 727-743. https://doi.org/10.1177/000348946207100313. PMID: 13974856.*

Minor concerns
In Figures 7 and 8, the number of mitochondria is evaluated. How were the findings and evaluation of ballooning? Since it is unclear which structures are mitochondria, please mark them with arrows or other symbols.

Response: We would like to apologize for the ambiguity in the figures and thank the reviewer for the recommendation. Ballooning and damaged cristae are indicators of mitochondrial dysfunction and oxidative stress^6,7^, which were observed in the SGNs and ANFs of advanced-aged mice in our study. We have modified the images and figure legends to clarify the mitochondrial structure in comparison to that of young mice and have revised the sentences in the Discussion section to incorporate these points (P.36, L.645-647).

*Reference #6: Miwa T, Katsuno T, Wei FY, Tomizawa K. Mitochondrial alterations in the cochlea of Cdk5rap1-knockout mice with age-related hearing loss. FEBS Open Bio. 2023; 13(7): 1365-1374. https://doi.org/10.1002/2211-5463.13655. PMID: 37258461.*

*Reference #7: Kamogashira T, Hayashi K, Fujimoto C, Iwasaki S, Yamasoba T. Functionally and morphologically damaged mitochondria observed in auditory cells under senescence-inducing stress. NPJ Aging Mech Dis. 2017; 3: 2. https://doi.org/10.1038/s41514-017-0002-2. PMID: 28649420.*

Reviewer #2: 
1. The sample size for cochlear histological analysis (n=3) was insufficient and had to be increased to at least 5 to ensure reliable and statistically robust data.

Response: Thank you for this insightful comment. We agree with the reviewer’s comment that a larger sample size yields more reliable results from the statistical analysis. Regrettably, restrictions on distributing the same strain in our country and the difficulty in maintaining mice near the end of their lifespan made it impossible to add specimens to the current study. We would like to mention that the calculated sample size to yield >0.8 power to detect significant differences with an alpha of 0.05, showed that the number of specimens we adopted was acceptable in the survival percentage of IHC, OHC, synapses, and SGN in the advanced-aged group, referring to a previous report using the same strain^1^. This description has been added to the Limitations section of the revised manuscript. (P.39, L.697-L.708)

*Reference #1: Sergeyenko Y, Lall K, Liberman MC, Kujawa SG. Age-related cochlear synaptopathy: an early-onset contributor to auditory functional decline. J Neurosci. 2013; 33(34): 13686-13694. https://doi.org/10.1523/JNEUROSCI.1783-13.2013. PMID: 23966690.*

2. The observed significant atrophy of the stria vascularis in aged CBA/CaJ mice raises the question of whether this morphological change affects the endocochlear potential (EP). The authors should discuss this potential implication or provide relevant data if available.

Response: Thank you for this comment. We agree that there is a concern about the relationship between morphological changes in SV and EP reduction. We were unable to evaluate and provide EP data in the current study because of technical limitations. Additional studies also demonstrate the coexistence of atrophy and reduced ion channel expression in the SV, as well as the decline in EP in aged CBA/CaJ mice^8,9^, indicating that morphological changes in the SV affect EP. Therefore, we have added these citations and revised the sentence in the Limitations section (p.38, L.677-679).

*Reference #8: Ding B, Walton JP, Zhu X, Frisina RD. Age-related changes in Na, K-ATPase expression, subunit isoform selection and assembly in the stria vascularis lateral wall of mouse cochlea. Hear Res. 2018; 367: 59-73. https://doi.org/10.1016/j.heares.2018.07.006. PMID: 30029086.*

*Reference #9: Liu T, Li G, Noble KV, Li Y, Barth JL, Schulte BA, et al. Age-dependent alterations of Kir4.1 expression in neural crest-derived cells of the mouse and human cochlea. Neurobiol Aging. 2019; 80: 210-222. https://doi.org/10.1016/j.neurobiolaging.2019.04.009. PMID: 31220650.*

3. The study measured reactive oxygen species (ROS) levels only in blood. Measuring ROS levels specifically within the cochlear tissue is crucial, particularly given that blood ROS levels may not accurately reflect ROS levels within the target organ/tissue. The authors should justify this omission or include cochlear ROS data.

Response: Thank you for this insightful comment. We agree that we should consider the ROS levels in the cochlear tissue, not just in the blood. Unfortunately, we were unable to measure the cochlear ROS data owing to technical limitations.

A previous study reported that mitochondrial aminoacyl-tRNA synthetase-KO mice showed elevated reactive oxygen species levels, activation of the mitochondrial apoptotic pathway, and morphologically abnormal mitochondria in cochlear hair cells^2^. Another study demonstrated that the enzyme catalyzing the deposition of 2-methylthio modifications in mt-tRNA KO mice showed a progressive accumulation of oxidative stress related to HC loss, SGN loss, and mitochondrial morphological changes^3^. These histological deformations were also observed in the current study, including a reduction in the mitochondrial number in the SGN soma and axons, as well as mitochondrial malformations, such as ballooning and damaged cristae. Therefore, we considered that a potentially high level of oxidative stress exists in the cochlea along with a systemically high level of ROS. We have added this description to the Discussion section. (P.37, L.661-667)

*Reference #2: Xu P, Wang L, Peng H, Liu H, Liu H, Yuan Q, et al. Disruption of Hars2 in Cochlear Hair Cells Causes Progressive Mitochondrial Dysfunction and Hearing Loss in Mice. Front Cell Neurosci. 2021; 15: 804345. https://doi.org/10.3389/fncel.2021.804345. PMID: 34975414.*

*Reference #3: Miwa T, Wei F-y, Tomizawa K. Cdk5 regulatory subunit-associated protein 1 knockout mice show hearing loss phenotypically similar to age-related hearing loss. Molecular Brain. 2021; 14(1). https://doi.org/10.1186/s13041-021-00791-w.*

4. The study exclusively used female mice. Consequently, the findings may not be representative of male animals. The title of the manuscript should explicitly state that the study was conducted on female mice (e.g., "...in Female CBA/CaJ Mice").

Response: We agree with the reviewer’s comment and we have revised the title of this article according to the reviewer’s suggestion.

5. In all figures presenting comparative data (young vs. aged), the data for young animals should be placed before the data for aged animals. This arrangement provides a more logical progression for readers.

Response: Thank you for this constructive comment. We have rearranged the figures according to the reviewer’s suggestion.

6. The manuscript requires thorough editing by a native English-speaking professional. Numerous grammatical and spelling errors are present. For example, on line 269, "10 ears of both groups…" should be written as "Ten ears…", consistent with standard English writing conventions for numbers at the beginning of a phrase. Careful proofreading is essential to improve clarity and professionalism.

Response: We sincerely apologize for the grammatical and spelling errors that were insufficiently corrected. The manuscript has been revised through professional proofreading to ensure clarity and accuracy.

Reviewer #3:
General:
1) One of the major conclusions by the authors is that environmental noise may be a driving factor in the underlying pathological changes in aged mice. I do not believe that this finding is fully supported by the data. Data from OHCs show that OHC loss was greatest at the low frequency regions of the cochlea, but also that there was severe OHC loss at the 16-32 kHz regions as well. Synapse loss was greatest at the 5.6 kHz region, but was fully recovered at 8 kHz with similar levels of synapse loss at the 11-32 kHz frequency range. Auditory thresholds were not the most affected at low frequencies and exhibited general hearing loss. For wave I analysis, 5.6 kHz exhibited no significant decrease in amplitude or latency in older mice. The effect of environmental noise is discussed only in the context of OHC, axon density, and synapse loss without discussion of the numerous other metrics analyzed in this study.

Response: Thank you for this comment. We agree that our initial conclusion overstates the role of ambient sound in the cochlear pathology in advanced-aged mice. Our study was observational rather than interventional, which did not allow us to suggest a causal relationship. We have now revised the manuscript to clarify that ambient sound is considered a *possible* contributing factor, not a definitive cause, of pathological changes in the aged cochlea.

We have focused our discussion on OHC and synapse loss because these parameters have some precedent in the literature for being affected by long-term environmental sound exposure. In contrast, we aimed to avoid overestimating other cochlear metrics because of the absence of supporting previous reports on the long-term effects of environmental noise.

As the reviewer correctly pointed out, significant OHC loss was observed across the cochlea, whereas the peak OHC loss was at 11.3 kHz, and a relatively greater loss was observed at lower frequencies. This differs from the OHC loss patterns induced by band noise, which often affect high frequencies^10^. One possible explanation is that the ambient sound spectrum in our vivarium was biased toward lower frequencies within the audible range of the mouse. Furthermore, a previous report showed that quiet housing (<40 dB) preserved HCs^4^, whereas the ordinal animal care facility in the current study had an LZeq > 70 dB. Thus, we hypothesized that steady lifelong acoustic exposure contributes to age-related OHC degeneration associated with aging. We also acknowledge that OHC degeneration is likely multifactorial, with metabolic stress from aging being another key contributor^2^.
Additionally, the synapses between the IHC and peripheral axons of SGNs are highly vulnerable to long-term noise exposure^11^, even under relatively low sound levels, when it continues long term^12^. In our data, 5.6 kHz showed the greatest synaptic loss, while 8.0 kHz was preserved and declined again at higher frequencies. A previous study using long-term white band noise exposure reported synaptic loss in the 12–32 kHz range^12^; however, the frequency spectrum of ambient sound in the current study was biased toward lower frequencies. Thus, the increased vulnerability at 5.6 kHz and relative sparing at 8.0 kHz, the boundary area between 5.6 and 12-32 kHz, may be observed.

Based on these findings and the supporting literature, we hypothesized that subtraumatic environmental noise exposure could be a contributing factor to the age-related cochlear pathologies observed in this study.
Regarding the results of the auditory function test, frequency-specific threshold elevation at low frequencies was not observed, likely because auditory function reflects the combined effects of sensory, neural, and strial degeneration with aging, which broadens the entire cochlea. Thus, it is likely that a comprehensive threshold shift occurred.

We have added these points and revised the Discussion section of the manuscript (P.32, L.569-592, P.34, L.604-611).

*Reference #2: Xu P, Wang L, Peng H, Liu H, Liu H, Yuan Q, et al. Disruption of Hars2 in Cochlear Hair Cells Causes Progressive Mitochondrial Dysfunction and Hearing Loss in Mice. Front Cell Neurosci. 2021; 15: 804345. https://doi.org/10.3389/fncel.2021.804345. PMID: 34975414.*

*Reference #4: Tarnowski BI, Schmiedt RA, Hellstrom LI, Lee FS, Adams JC. Age-related changes in cochleas of mongolian gerbils. Hear Res. 1991; 54(1): 123-134. https://doi.org/10.1016/0378-5955(91)90142-v. PMID: 1917712.*

*Reference #10: Qi M, Gao Z, Qiu Y, Wang R, Tian K, Yue B, et al. NOX2 Contributes to High-Frequency Outer Hair Cell Vulnerability in the Cochlea. Adv Sci (Weinh). 2025: e08830. https://doi.org/10.1002/advs.202408830. PMID: 40569247.*

*Reference #11: Kujawa SG, Liberman MC. Adding insult to injury: cochlear nerve degeneration after "temporary" noise-induced hearing loss. J Neurosci. 2009; 29(45): 14077-14085. https://doi.org/10.1523/JNEUROSCI.2845-09.2009. PMID: 19906956.*

*Reference #12: Feng S, Yang L, Hui L, Luo Y, Du Z, Xiong W, et al. Long-term exposure to low-intensity environmental noise aggravates age-related hearing loss via disruption of cochlear ribbon synapses. Am J Transl Res. 2020; 12(7): 3674-3687. PMID: 32774726.*

2) The authors missed the opportunity to compare across metrics in the same cohort of mice. For example, audiometric threshold is not compared with any structural measure in this study. It would be interesting to see how in the aged mice, number of synapses correlated with wave I amplitude or latency. Furthermore, why were ABRs only collected from 5 mice in each group? How many of the ears for histology had audiometric data associated with them? Without the study of associations between metrics, the conclusions of this study are not strong.

Response: We would like to thank the reviewer for the insightful and constructive comment. We agree that comparing audiometric data with cochlear structural measures could strengthen our conclusions. However, due to the small sample size, particularly in the aged group, we were limited in our ability to robustly analyze the correlations between auditory function and histological damage within each cohort. Regarding audiometric data from the ears used for histological evaluation, both ears from all of the animals subjected to ABR testing were utilized for either whole-mount or cross-sectional histology because different fixation procedures were required for immunofluorescence and electron microscopy, and we aimed to reduce the total number of animals sacrificed.

Nevertheless, we have performed a correlation analysis using data from the combined young and aged groups to assess the relationship between synapse counts and ABR wave I amplitude and latency, in response to the reviewer’s comment. This analysis has revealed a significant correlation between the synapse count and wave I amplitude at several frequencies. This finding provides important support for our discussion of the relationship between cochlear synaptic integrity and ABR metrics. We have added descriptions of these correlation analyses to the Methods (P.15, L.267-269) and Results (P.18, L.331-P.19, L.335), and revised the Discussion section (P.29, L.527-529).

3) When reporting the statistics, please report the results of the 2-way ANOVA, not just the post-hoc Sidak test. Before reporting the Sidak test, please report the F and values for the interaction between predictor variables at minimum. Without this information, it is impossible to know if the post-hoc tests are appropriate.
Response: We thank the reviewer for this insightful comment. We agree with the reviewer's suggestion that it is reasonable to include the overall results in the statistical description. We apologize for the lack of a description of the results and have added F and *p* values to the manuscript.

4) The discussion of this study suffers from lack of a thorough review of previously published literature to place these findings in context. A few examples: First, the cited study on line 477 is from induced SGN loss in a single cat. More information is needed here, as there are numerous studies showing that aging results in SGN loss that contributes to elevated hearing thresholds (See Frisna et al., 2016, Age-related hearing loss: prevention of threshold declines, cell loss and apoptosis in spiral ganglion neurons). Second, there are no citations supporting the statement that thresholds could reflect HC loss and SV atrophy (line 479) , even though there are many studies examining the exact relationship between loss in these areas and age-related hearing loss (see Stebbins et al., 1979 or Lang et al., 2010).

Response: We thank the reviewer for pointing this out and providing a set of references. We apologize for the insufficient literature review and the lack of appropriate references. As recommended by the reviewer, we have revised the manuscript and added citations to the Discussion section. (P.28, L.513-P.29, L.517, P.29, L.523-524)

Abstract:
Line 36-37: Please also state the number of young mice used.

Response: We thank the reviewer for pointing out the lack of relevant information, which has now been added.
Line 38- Please clarify what is meant by frequency characteristics of environmental sound.
Line 39- This is not accurate, as the environmental sound peaked at 1 kHz with a tapered decrease in intensity to around 10 kHz. It would be more accurate to just synapses, as loss of OHC’s and synapses were observed at all frequency regions (with OHC loss peaking at 11.3 kHz region) and synapse loss peaking at the 5.6 kHz region.

Response: Regarding the above two concerns, we would like to apologize for the ambiguity and inaccuracy in the description. What we intended to mention here was that the dominant frequency of OHC and synaptic loss corresponded to the distribution of dominant sound pressure level in the frequency domain of the ambient sound. We appreciate the reviewer’s constructive comments and revised the sentence as follows:

“The loss of outer hair cells peaked at 11.3 kHz, and the greatest synapse loss was observed in the 5.6 kHz region, which was covered by the dominant frequency in the ambient sound.”

Introduction:
Line 58- Please use a different citation for the negative effect of tinnitus on quality of life, as the cited study just looks at incidence, not disease burden.

Response: We apologize for these inappropriate citations. We have replaced it with paper^13^ reporting a study that demonstrated that tinnitus severity was associated with a lower quality of life score in the older adult population.

*Reference #13: Nondahl DM, Cruickshanks KJ, Dalton DS, Klein BE, Klein R, Schubert CR, et al. The impact of tinnitus on quality of life in older adults. J Am Acad Audiol. 2007; 18(3): 257-266. https://doi.org/10.3766/jaaa.18.3.7. PMID: 17479618.*

Line 71- Please use full CBA/CaJ strain label here.

Response: We apologize for the lack of full strain labeling. This has been corrected accordingly.

Materials and Methods:
Line 87- Were these mice housed in the same environment for the duration of their lifespans?

Response: Aged mice were kept in an identical environment until they were shipped to the experimental facility. To clarify this, we revised the sentence as follows: (P.5, L.94-95)

Line 92-95- Were these recordings taken at multiple time points during the aged mouse lifespan?

Response: We repeated the sound measurements during the lifespan of the aged mice, and the results were identical. This information has been added to the Methods section. (P.5, L. 98-P.6, L.100)

Line 97-98- Were the young animals shipped for experiments as well?

Response: We apologize for the ambiguity in the manuscript. Young animals were also shipped, as has been added to the sentence. (P.6, L.100)

Line 116- Were the ABR stimuli presented monaurally? Or binaurally? Was the reference electrode on the same side for each mouse?

Response: In our measurement procedure, the ABR stimuli were presented monaurally and the reference electrode was on the median of the vertex. We have revised this sentence to clarify the measurement setup. (P.7, L.123-124)

Line 121-123- What was the starting dB value? How could sound levels be increased from <20 dB below threshold if the threshold of the animal was not determined yet?
Line 122- should be ‘=80 dB SPL’, as the measurements of wave I amplitude at latency were performed with data from 80 dB SPL stimuli.

Response: Regarding the above two concerns, we would like to apologize for the uncertainty in the description. We measured the ABR response evoked by the stimuli at exactly 20–80 dB SPL with 5 dB steps, not related to the threshold of the individual ear. Additionally, as the reviewer noted, 80 dB SPL stimuli should have been included at the stimulus levels. The sentence has been revised accordingly. (P.7, L.126)

Line 123-125- Were reviewers of the waveforms blinded to age group?

Response: We apologize for the lack of detail. Yes, blinded reviewers for group labeling evaluated the ABR waveforms, which have been included in the Methods section. (P.7. L.131)

Line 163-167- Please clarify the difference in these fluorescent microscopes. Why were two different microscopes used?
Response: We used a fluorescent microscope (BZ-X700) and a fluorescent confocal microscope (TCS SP8) to capture the cochlear immunofluorescent images. The BZ-X700 has a fast image-capturing speed and sufficient resolution quality for the quick review of cochlear whole mounts, making cochlear frequency maps, and differentiating HCs to be counted. Confocal microscopy provides magnified images with a higher resolution, enabling the evaluation of cochlear synapses. The sentence in the Methods section was revised to clarify this point. (P.9, L.169)

Line 203-204- It is not clear how missing HC’s were determined. The authors state that the lack of Myo7a-positive cell bodies was used as the criterion for missing HC’s, but was there another metric used? As it is presented, it is impossible to quantify that something is missing based on the fact that Myo7a is missing, as there is no way to confirm that it was expressed in that region originally. Another approach to this would be to normalize the percent survival of HC’s in aged mice by normalizing it to HC’s in young mice.

Response: We thank the reviewer for the crucial and insightful comments and apologize for the inaccuracy of our measurements. As the reviewer pointed out, we could not use an alternative metric, such as phalloidin staining, to detect missing HC, which led to an insufficient calculation of the HC survival rate. Therefore, we adopted a normalization process to assess HC survival in the aged group. For normalization, the number of surviving IHCs/OHCs at each specific frequency from individual advanced-aged mouse ears was divided by the average number from young mice. These results are consistent with previous results, which showed the peak of OHC loss at 11.3 kHz, and the survival rate tended to be lower at 5.6 and 8.0 kHz than at high frequencies.

Thus, the Methods (P.11, L.204-207) and Results (P.17, L.315-P.18, L.322) in the manuscript and Figure 3 have been revised based on this modified analysis.

Line 240- Where 100 axons from the same mice? Or 100 axons within the age group across multiple mice?

Response: We apologize for the ambiguity in the manuscript. One hundred axons per ear were evaluated. This sentence has been revised for clarity. (P.13, L.241)

Line 249-251- How many samples were taken from each age group? Were these the same mice used for audiometric and histological analysis?

Response: We have used five samples from each age group, which have been added to the manuscript. (P.14, L.253) Unfortunately, the mice utilized for the whole blood measurements were not the same as those used for audiometric and histological analysis. However, the subjects used for the d-ROM measurements should have comparable systemic conditions, given their identical age, sex, and raised conditions.

Line 261- please add the specific methods used to normalize data to the young group. Also, please specifically state which analyses used normalized data, as this is important information for interpreting the data presented.

Response: We apologize for the lack of details in the statistical description. The measured values were normalized by dividing the raw individual data by the average for the young group. This description has been added to the Methods section, and we have listed the analyses used for the normalized data. (P.15, L.263-267)

Lines 258-260- Please include the specific predictor variables used for the 2-way ANOVAs. From context in the results, there are frequency and age, but they need to be stated.

Response: Thank you for pointing this out. Following the reviewer’s suggestion, we have clarified the variables as follows:

“We performed a two-way analysis of variance (ANOVA) with frequency and age as independent variables, followed by Šidák’s post-hoc test to analyze statistical differences in DPOAE and ABR measurements, HCs and synaptic counts, SVA, SMA, SGNs, and ANFs measurements.”

Results
Figure 2- ABR thresholds in older mice at 5.66 kHz were above the 80 dB SPL level used to calculate P1 amplitude and P1 latency. Does this mean that data from a subset of the aged mice was reported in 2C and 2D? How did the authors account for mice with thresholds above the 80 dB stimulation used for suprathreshold ABR analysis?

Response: We apologize for the ambiguity in the procedures used in our analyses. The plots of the ABR threshold are shown as mean ± SEM, and several subjects exhibited a threshold value of just 80 dB SPL. We measured P1 amplitude and latency in these ears and incorporated them into the analysis. Therefore, we were unable to collect and include data from ears with thresholds > 80 dB SPL. In order to clarify this process, we added the following sentence to the Methods section:

“The ABR P1 metrics of frequencies with an 85 dB SPL threshold for each ear were excluded from the analysis.” (P.7, L.135-P.8, L.137)

Line 318- Please replace the word ‘huge’ with more accurate language, such as ‘greatest loss’.

Response: We apologize for the inappropriate term. This has been corrected as follows.

“The proportion of synaptic loss was greatest at 5.6 kHz.”

Figure 4a- Although stated in the caption, please add a legend for the antibodies used for the immunohistochemical images.

Response: We apologize for the missing legends for the antibodies in the images, which have been added.

Figure 4b- Do the authors have any hypothesis why synapses at 8 kHz were minimally affected? Also, please change the y-axis to a percentage, similar to the y-axes in figure 3b/c

Response: Thank you for your insightful comment. A previous report demonstrated heightened synaptic loss in the region of 30–70% of the cochlear turn in mice exposed to low-intensity white noise for a long duration (3 months)^12^, suggesting that the audible spectrum of mice corresponding to 12–32 kHz^14^ can be vulnerable to prolonged acoustic exposure concerning synaptic degeneration. In this regard, the ambient sound measured in the current study exhibited a frequency profile with a peak shifted toward lower frequencies; thus, it is possible that synaptic loss was more pronounced at 5.6 kHz, and 8 kHz, the boundary area between 5.6 and 12-32 kHz, was minimally affected. We have now added this description to the Discussion section. (P.34, L.604-611)

Additionally, we changed the y-axis unit in Figure 4B, following the reviewer’s recommendation.

*Reference #12: Feng S, Yang L, Hui L, Luo Y, Du Z, Xiong W, et al. Long-term exposure to low-intensity environmental noise aggravates age-related hearing loss via disruption of cochlear ribbon synapses. Am J Transl Res. 2020; 12(7): 3674-3687. PMID: 32774726.*

*Reference #14: Viberg A, Canlon B. The guide to plotting a cochleogram. Hear Res. 2004; 197(1-2): 1-10. https://doi.org/10.1016/j.heares.2004.04.016. PMID: 15504598.*

Line 349-351- These data should be frames slightly differently to be more understandable. Although p=0.052 is not significant, it is a trend toward significance. A statement should be made toward this end, stating that although the effect size is large, the null hypothesis cannot be rejected. Furthermore, reporting of the 2-way ANOVA results would be helpful here, as it would determine if reporting the post-hoc comparisons between ages at the apical turn are appropriate.

Response: Thank you for this constructive comment. The statement of the results in this section has been revised, and the results of the two-way ANOVA (F and p values for the interaction) have been added. (P.21, L.373-375) Although the *p*-value for the interaction is not statistically significant, we believe that the result of the post-hoc test should be considered, as the aim of the analyses in the current study was to focus on intergroup comparisons at each frequency (cochlear turn).

Discussion
Line 483- Please add relevant citations showing the known relationship between OHC loss and elevated DPOAE thresholds.

Response: We have added a previous report^15^ stating that OHC loss can cause DPOAE threshold shifts (P. 29, L.523-524). This study showed that OHC loss and elevated DPOAE thresholds were within normal hearing thresholds.

*Reference #15: Davis B, Qiu W, Hamernik RP. Sensitivity of distortion product otoacoustic emissions in noise-exposed chinchillas. J Am Acad Audiol. 2005; 16(2): 69-78. https://doi.org/10.3766/jaaa.16.2.2. PMID: 15807046.*

Line 487- Please change this citation to one that directly examines the relationships between auditory nerve activity and ABR wave I metrics.

Response: We apologize for these inappropriate citations. We replaced this with several papers that describe or explore the relationship between ABR wave 1 amplitude, SGN number, and synchrony of firing^16-18^, as well as ABR wave 1 latency, synaptic transmission, and myelination of ANFs^18,19^.

*Reference #16: Zhang ZJ, Guan HX, Yang K, Xiao BK, Liao H, Jiang Y, et al. Estimation of the status of spiral ganglion neurons and Schwann cells in the auditory neural degeneration mouse using the auditory brainstem response. Acta Otolaryngol. 2018; 138(7): 603-609. https://doi.org/10.1080/00016489.2018.1436766. PMID: 29553844.*

*Reference #17: Davis RL, Crozier RA. Dynamic firing properties of type I spiral ganglion neurons. Cell Tissue Res. 2015; 361(1): 115-127. https://doi.org/10.1007/s00441-014-2071-x. PMID: 25567109.*

*Reference #18: Rutherford MA, Chapochnikov NM, Moser T. Spike encoding of neurotransmitter release timing by spiral ganglion neurons of the cochlea. J Neurosci. 2012; 32(14): 4773-4789. https://doi.org/10.1523/JNEUROSCI.4511-11.2012. PMID: 22492033.*

*Reference #19: Jyothi V, Li M, Kilpatrick LA, Smythe N, LaRue AC, Zhou D, et al. Unmyelinated auditory type I spiral ganglion neurons in congenic Ly5.1 mice. J Comp Neurol. 2010; 518(16): 3254-3271. https://doi.org/10.1002/cne.22398. PMID: 20575058.*

Line 489-495- In this section the authors link loss of SGNs to reduced ABR wave I amplitudes. Please provide citations to published literature examining this relationship and further citations studying the relationship between HC synapses and wave I amplitude.

Response: We apologize for the lack of relevant citations. As the reviewer has pointed out, the relationship between HC synapses and wave I amplitude is also a substantial perspective for discussion. We have added citations for the link between SGN and synapse loss and ABR wave I amplitude^11, 16^ and have incorporated the relevant sentences into the manuscript. (P.30, L.533-538)

*Reference #11: Zhang ZJ, Guan HX, Yang K, Xiao BK, Liao H, Jiang Y, et al. Estimation of the status of spiral ganglion neurons and Schwann cells in the auditory neural degeneration mouse using the auditory brainstem response. Acta Otolaryngol. 2018; 138(7): 603-609. https://doi.org/10.1080/00016489.2018.1436766. PMID: 29553844.*

*Reference #16: Kujawa SG, Liberman MC. Adding insult to injury: cochlear nerve degeneration after "temporary" noise-induced hearing loss. J Neurosci. 2009; 29(45): 14077-14085. https://doi.org/10.1523/JNEUROSCI.2845-09.2009. PMID: 19906956.*

Line 497- Are there published studies of orphan synapses in aging or is this the first to document the minimal change in orphan synapses with aging?

Response: A previous study reported the presence of orphan ribbons in aged CBA/CaJ mice^20^ and found that the number of orphan ribbons was elevated during the basal turns of aged mice. This differs from our findings, which show minimal changes in orphan ribbons. We have assumed that older age could be one of the factors causing this inconsistency, as another study reported that presynaptic ribbons progressively disappeared after postsynaptic density degeneration due to SGN loss^21^. We have added these references and revised the manuscript accordingly (P.30, L.549-P.31, L.552).

*Reference #20: Okur MN, Sahbaz BD, Kimura R, Manor U, Patel J, Park JH, et al. Long-term NAD+ supplementation prevents the progression of age-related hearing loss in mice. Aging Cell. 2023; 22(9): e13909. https://doi.org/10.1111/acel.13909. PMID: 37395319.*

*Reference #21: Yuan Y, Chi F. Dynamic changes in hair cell ribbon synapse induced by loss of spiral ganglion neurons in mice. Chin Med J (Engl). 2014; 127(10): 1941-1946. PMID: 24824260.*

Line 517-519- The middle of the cochlea does not correspond to low frequencies.

Response: We would like to apologize for the ambiguity of the sentence, which was intended to express that the greatest OHC loss was shown at the middle frequency, and the intensity of the OHC loss at the lower frequencies was relatively greater than that at high frequencies. This sentence has been clarified as follows:

“The most significant OHC degeneration observed in the current study occurred in the mid-frequency cochlear region. Furthermore, the intensity of OHC loss was greater at lower frequencies than at higher frequencies.” (P.32, L.569-572)

Line 538-539- It is not accurate to say that the environmental noise was in the low to middle frequencies of the mouse audible range, as the mouse audible range is from 1-1000 Hz with the highest sensitivity at 16 kHz (Reynolds et al., 2010). It would be more accurate to say these frequencies are at the lower range.

Response: We apologize for the inaccuracy in the manuscript and agree with the reviewer’s comment. The sentence has been revised as follows, and citation^22^ has been added:

“The high proportion of ambient sound pressure in our vivarium (Fig 1) was dominated by lower frequencies (5.6, 8.0, and 11.3 kHz) within the audible range for mice (1 to 100 kHz).” (P.33, L. 590-592)

*Reference #22: Reynolds RP, Kinard WL, Degraff JJ, Leverage N, Norton JN. Noise in a laboratory animal facility from the human and mouse perspectives. J Am Assoc Lab Anim Sci. 2010; 49(5): 592-597. PMID: 20858361.*

569- This line states a 60% SGN survival, while line 478 states at 50% SGN survival. This information should be in the results as well.

Response: We apologize for the inconsistent description. The SGN survival percentage in the aged group has been added to the Results section following the reviewer’s suggestion (P.24, L.428-430). The percentage in line 478 has now been revised to 60% (P.29, L.517).

Conclusion
Line 630-632- These results do not suggest that the severe SGN loss is due to mitochondrial damage and stress, just that aged mice exhibit oxidative stress. At minimum, correlational analysis between these metrics would need to be done for this statement to be included.

Response: Thank you for your insightful comments. We agree with the reviewer’s comment regarding the lack of correlation analysis in the current study. However, we observed the coexistence of SGN loss and potential damage resulting from oxidative stress, which was reflected in mitochondrial loss, malformations, and other morphological abnormalities in the cytoplasm of the SGNs. Furthermore, we demonstrated systemic oxidative stress levels by measuring ROS levels directly in aged mice. Therefore, we have revised the sentence to emphasize that concurrent severe SGN loss, mitochondrial damage, and systemic oxidative stress elevation were observed in advanced-aged mice and refrained from insisting on causality as follows:

“Severe loss of SGNs in advanced-aged mice may occur due to primary neural degeneration, and the coexistence of mitochondrial damage and systemic oxidative stress indicates metabolic dysfunction in SGNs.” (P.40, L.718-721)

Reviewer #4: 
Major:
1. The numbers of animals used for many aspects of this study are too few to draw reasonable conclusions. Many of the negative findings (e.g., the lack of differences in myelination) are grossly underpowered (n=3 per group) and even when differences are observed, the n’s are often too small to have a confident estimate of the difference between groups.

Response: Thank you for your insightful comment. We agree with the reviewer’s comment that a larger sample size yields more power and reliable results from the statistical analysis. Regrettably, restrictions on distributing the same strain in our country and the difficulty in maintaining mice near the end of their lifespan made it impossible to add specimens to the current study. We would like to mention that the calculated sample size to yield >0.8 power to detect significant differences with an alpha of 0.05, showed that the number of specimens we adopted was acceptable in the survival percentage of IHC, OHC, synapses, and SGN in the advanced-aged group, referring to a previous report using the same strain^1^. This description has been added to the Limitations section of the revised manuscript. (P.39, L.697-708)

*Reference #1: Sergeyenko Y, Lall K, Liberman MC, Kujawa SG. Age-related cochlear synaptopathy: an early-onset contributor to auditory functional decline. J Neurosci. 2013; 33(34): 13686-13694. https://doi.org/10.1523/JNEUROSCI.1783-13.2013. PMID: 23966690.*

2. Along the same lines, there are instances were non-independent data are analyzed with ANOVA, which assumes independent samples. For example, in the hair cell counts, in some cases two “ears” come from one animal (and are therefore not independent) and other individual “ears” are only from one animal. Adjustment to the statistical plan should be made.

Response: We thank the reviewer for the comment regarding the statistical analysis and independence of the samples. We agree that ANOVA assumes independence among samples; however, in our study, we included both ears from the same animals. Because of the distinct fixation and processing procedures required for immunofluorescence versus electron microscopy assessments, it was not feasible to use both ears from the same animal for separate evaluations (i.e., we were unable to use one ear for immunofluorescence and the other for electron microscopy). Additionally, to strictly adhere to ethical principles, specifically to minimize the number of animals sacrificed, we utilized both ears from a single animal within the same evaluation method and group rather than doubling the number of animals sacrificed.

We acknowledge that the incomplete independence of statistical analysis is a crucial limitation of our study. Thus, this description has been added to the Limitations section. (P.39, L.698-703)

3. The absence of an intermediate age group is a major weakness which limits our understanding of when these changes occur and the degree to which the findings in the young group represent a developmental trajectory.

Response: We thank the reviewer for the insightful and constructive comment. We agree that including an intermediate age group would have provided a more comprehensive understanding of the progression of age-related cochlear degeneration. A previous study by Sergeyenko et al. investigated the auditory function and cochlear histological changes, such as HCs, synapses, and SGNs, across multiple age groups in CBA/CaJ mice^1^. Our study evaluated additional aspects of cochlear pathology, including the stria vascularis, cochlear hydrops, myelination of ANFs, and mitochondrial morphology, to comprehensively assess naturally advanced senescent mice. However, as the reviewer has pointed out, the lack of intermediate age groups limits our study’s ability to determine both the precise onset and trajectory of these degenerative changes. We have added this description to the limitations section. (P.38, L.680-685)

*Reference #1: Sergeyenko Y, Lall K, Liberman MC, Kujawa SG. Age-related cochlear synaptopathy: an early-onset contributor to auditory functional decline. J Neurosci. 2013; 33(34): 13686-13694. https://doi.org/10.1523/JNEUROSCI.1783-13.2013. PMID: 23966690.*

4. The argument that ambient noise in their vivarium (which does seem rather loud) is responsible for hearing loss is not compelling. The ambient noise is quite low frequency and does not align well to the distribution of hearing loss, which is mostly uniform in these mice.

Response: Thank you for this insightful comment. We agree that the ambient sound in our vivarium was relatively high, with the spectrum biased toward lower frequencies. As the reviewer pointed out, the distribution of hearing loss was uniform in the elderly group. We assumed that auditory function reflects the combined effects of sensory, neural, and strial degeneration with aging and broadened pathology in the whole cochlea, which may have contributed to the absence of a frequency-specific threshold elevation at low frequencies.

We would also like to mention our speculation on the relationship between the ambient sound spectrum and cochlear pathology, especially the distribution of OHC and synaptic loss. Significant OHC loss was observed across the cochlea, with the peak OHC loss occurring at 11.3 kHz. A greater loss was observed at lower frequencies than at higher frequencies. This differs from the OHC loss patterns induced by band noise, which often affect high frequencies^10^. One possible explanation is that the ambient sound spectrum in our vivarium was biased toward lower frequencies within the audible range of the mouse. Additionally, synapses are highly vulnerable to noise exposure^11^, even under relatively low sound levels, when exposure continues over a long term^12^. In our data, 5.6 kHz showed the greatest synaptic loss, while 8.0 kHz was preserved and declined again at higher frequencies. A previous study using long-term white-band noise exposure reported synaptic loss in the 12–32 kHz range^3^; however, the frequency spectrum of ambient sound in the current study was biased toward lower frequencies. Thus, the increased vulnerability at 5.6 kHz and relative sparing at 8.0 kHz, the boundary area between 5.6 and 12-32 kHz, may be observed.

Therefore, we have assumed that subtraumatic environmental noise exposure could contribute to the age-related cochlear pathologies observed in this study. We have added these points and revised the Discussion section of the manuscript (P.32, L.569-572, P.33, L.587-L.594, P.34, L.604-611).

*Reference #10: Qi M, Gao Z, Qiu Y, Wang R, Tian K, Yue B, et al. NOX2 Contributes to High-Frequency Outer Hair Cell Vulnerability in the Cochlea. Adv Sci (Weinh). 2025: e08830. https://doi.org/10.1002/advs.202408830. PMID: 40569247.*

*Reference #11: Kujawa SG, Liberman MC. Adding insult to injury: cochlear nerve degeneration after "temporary" noise-induced hearing loss. J Neurosci. 2009; 29(45): 14077-14085. https://doi.org/10.1523/JNEUROSCI.2845-09.2009. PMID: 19906956.*

*Reference #12: Feng S, Yang L, Hui L, Luo Y, Du Z, Xiong W, et al. Long-term exposure to low-intensity environmental noise aggravates age-related hearing loss via disruption of cochlear ribbon synapses. Am J Transl Res. 2020; 12(7): 3674-3687. PMID: 32774726.*

5. Were the blood samples taken from the same mice that hearing was measured in? If so, it would be important to calculate correlations between hearing metrics and this marker of oxidative stress.

Response: Thank you for this insightful comment. We agree that the correlation between hearing metrics and oxidative stress markers is significant. Blood samples used to measure d-ROMs were collected from a separate cohort of mice that were strictly matched to the hearing/histology cohort in terms of age, strain, and sex. Therefore, we were unable to perform correlation analyses for this dataset because the biochemical data and auditory phenotypes were obtained from different individuals. We have added this description to the limitations section. (P.38, L.688-690)

Additionally, the number of animals used for the d-ROM measurements was not included in the total number of animals in the manuscript. We have now revised the manuscript to provide a more rigorous description.

Minor:
1. There are many typos, grammatical errors and awkward word choices. I have outlined a few here. A thorough editing is needed.

Response: We sincerely apologize for the typographical and grammatical errors that were insufficiently corrected and the use of inappropriate terms. We thank the reviewer for the suggestions regarding these mistakes. This has been corrected in response to the reviewers’ comments and the revised manuscript has undergone professional proofreading to ensure clarity and accuracy.

2. There is a run-on sentence on lines 32-34

Response: The sentence has been clarified as follows:

“In this study, we investigated the audiological physiology and cochlear pathology of advanced-aged CBA/CaJ mice, a mouse strain that resists early pathological hearing loss. The subjects naturally aged close to their lifespan limit (over two years) under normal vivarium conditions.”

3. The age of the young mice should be listed in the abstract

Response: We thank the reviewer for pointing out the lack of relevant information, which has now been added.

4. Delete the comma after “area” in line 38
5. Line 46 – change “due to” to “potentially associated with”
6. Line 83 – change “keeping environment” to something like “laboratory animal environment” or “vivarium”
7. Space between no. and 21051 in line 100.
8. Line 104 – change “noise” to “ambient sound”

Response: Five suggestions (#4–8) have been corrected following the reviewer’s recommendation.

9. SV and SM abbreviations are not used consistently. Please define them once and use throughout.

Response: We have corrected these abbreviations consistently. Furthermore, ‘SVA’ and ‘SMA’ have been revised to ‘SV area’ and ‘SM area’ to avoid confusion.

10. “Huge” is not an appropriate term to describe data. This word is used 3x. Please find another word.

Response: We apologize for the inappropriate use of these words. “Huge” has been corrected to “large.”

11. Line 249 – delete “blood”

Response: This has been corrected following the reviewer’s suggestion.

12. Please state how blood was collected. How long was blood left to sit before supernatant was collected?

Response: We apologize for the lack of procedural details regarding blood collection. Blood samples were collected transcardially and centrifuged immediately (within 2 min of sampling). This statement has been added to the Methods section. (P.14, L.254-255)

13. Fig 5 – please label SM and SV

Response: The figures and legends have been revised according to the reviewer’s suggestion.

14. In figs where aged data is normalized to young data (eg Fig 5), please indicate if the aged findings were normalized to the average young value.
Response: A statement regarding the normalized data by the young group has been added to the figure legends for Figs. 3 and 4.

15. The phrase “raising environment” is not right. Perhaps “vivarium” is better.

Response: This has been corrected following the reviewer’s suggestion.

Reviewer #5: (No Response)

The author of this article studied the changes in inner ear structure and auditory function in an animal model of age-related hearing loss. Although this study is not a intervention study and the innovation was limited, the research content has certain value. I have some questions as follows.

1. Please mark the references in the first line of page 4 of the manuscript.

Reference: The reference^23^ has been added to the manuscript.

*Reference #23: WorldHealthOrganization. 10 facts on ageing and health 2017 May 1 [cited 2025 July 16]. Available from: https://who-dev5.prgsdev.com/m/news-room/fact-sheets/detail/10-facts-on-ageing-and-health.*

2. Please use signs such as arrows to indicate the relevant and explanatory information described in the legend text of Figures 3A and 7B.

Response: We would like to apologize for the lack of clarity in the figures. To clarify these figures, arrows and abbreviations have been added to indicate specific structures.

3. The statement in the line 477-479 is incomprehensible. Please elaborate on this part in detail.

Response: We apologize for the unclear sentence. In this section, we describe whether SGN loss contributes to an ABR threshold shift in advanced-aged mice in the current study. The correlation between SGN loss and hearing loss is supported by a previous report^24, 25^, which demonstrated that less than 70-75% SGN loss does not affect hearing threshold elevation. To clarify this, we have revised the sentence as follows:

“However, several studies reported that only severe SGN loss was related to hearing threshold shifts. Schuknecht HF et al. demonstrated that hearing threshold elevation was only observed when SGN loss exceeded 75%. Frisna et al. detected a moderate correlation between SGN loss and hearing threshold in the aged mouse model, which required approximately 70% loss of SGNs. These literatures indicate that a 60% loss of SGNs in the current study would not mainly contribute to the ABR threshold shift.” (P.28, L.512-P.29, L.518)

*Reference #24: Schuknecht HF, Woellner RC. An experimental and clinical study of deafness from lesions of the cochlear nerve. J Laryngol Otol. 1955; 69(2): 75-97. https://doi.org/10.1017/s0022215100050465. PMID: 14354340.*

*Reference #25: Frisina RD, Ding B, Zhu X, Walton JP. Age-related hearing loss: prevention of threshold declines, cell loss and apoptosis in spiral ganglion neurons. Aging (Albany NY). 2016; 8(9): 2081-2099. https://doi.org/10.18632/aging.101045. PMID: 27667674.*
