# Supplementary material for: Histopathological change of age-related hearing loss in female advance-aged CBA/CaJ mice
Source: PLoS One. 2025 Oct 7;20(10):e0334021. doi: 10.1371/journal.pone.0334021 (PMC12503338; doi:10.1371/journal.pone.0334021)
Supplement: Attachment — Submitted filename: Rev2_Response to Reviewers.docx [file pone.0334021.s016.docx]

**Point to Point Response to the Reviewers**

Manuscript Number: PONE-D-25-21282R2

We would like to thank the editor and the reviewers for their comments. We have re-edited and improved our manuscript as suggested. In the following, we provide a point-to-point response to every comment raised. We have highlighted portions of the manuscript that have been updated in response to the reviewers’ comments.

Editor: 
1. Please provide ANOVAs per mouse (N=5), as well as per ear for functional data.

(…Mice (n = 10 ears of 5 animals per group) were anesthetized intramuscularly using ketamine (75 mg/kg)…)

Response: We performed two-way ANOVAs and post hoc tests using per-mouse analysis, in which the measured values from both ears of each mouse were averaged. The overall results were consistent with the per-ear analysis, except for ABR P1 latency at several frequencies, where statistical significance was not observed due to reduced power from the smaller effective sample size. We have added this description to the Methods section (P.15, L.269–273) and the Results section (P.17, L.312–315), as well as in the Supporting Information (S1 Fig and S2 Table).

2. Please amend phrase "three ears in both groups" (in Section Survival of cochlear HCs and synaptic ribbons; line 312 of manuscript with changes highlighted) to "three ears in two mice from both groups"

(Mice (n = 3 ears of 2 animals in each group) were euthanized... taken from line 148-149 of amended manuscript)

Response: This sentence was corrected following the editor’s recommendation. (P.18, L.330)

3. For EM analyses, please provide statistics per mouse and per ear.

(The mice (n = 8 ears of 4 animals per group) were anesthetized...)

Response: We performed two-way ANOVAs and post hoc tests in the per-mouse analysis, following the same approach used for the cochlear function test (Editor’s Comment #1). The results showed trends comparable to the per-ear analysis, with statistical significance detected in the same metrics. This description has been added to the Methods section (P.15, L.269–273) and the Results section (P.22, L.404–405; P.27, L.480–482), as well as in the Supporting Information (S3 Fig and S4 Table).

4. Please clarify, in the "Statistical analyses"section, how the data from citation 11 was used for calculations of sample size.

Response: We apologize for the insufficient detail in our original submission. A more detailed description has now been added to the manuscript (P.16, L.280–285).

Reviewer #3:
I want to thank the authors for the time taken to respond to all of the comments. There was a lot of work done on the paper that has significantly improved it. However, I still have a few concerns with this manuscript.

Response: We sincerely thank the reviewer for the careful reading of our manuscript and the constructive comments. The thoughtful feedback has greatly improved the quality and clarity of the work, and we are truly grateful for the time and effort devoted to this review.

In response to the additional concerns raised, we have carefully addressed each point and revised the manuscript accordingly. We believe these further revisions have strengthened the paper, and we hope that the changes satisfactorily resolve the remaining issues.

1. Please clarify why 20 dB SPL was the starting point for determining thresholds. Did no mice have thresholds below 20 dB SPL? If so, then this is inconsistent with the data presented in Figure 2 B, where the average threshold from young mice at 16 kHz is less than 20 dB. And if the loudest. Please make sure that your methods section is accurate.

Response: We apologize for the ambiguity in our previous description. In young mice, particularly at 16 kHz, robust ABR waveforms are often detected even at 20 dB SPL. In such cases, the ABR Peak Analysis Software automatically determines thresholds below 20 dB SPL, and we adopted these automatically calculated values as the final thresholds. To clarify this procedure, we have revised the Methods section (P.7, L.131–133).

2. Thank you for adding a correlation analysis between IHC synapses and ABR P1 amplitude and latency. However, I have a few concerns about the analysis and presentation of this data. First, please remove any regression lines that are not significant, as they do not mean anything and can cause the illusion of significance. Second, there is an inconsistency between the results reported in the text and the figure. The text states that IHC synapse counts at both 16 kHz and 22 kHz were significantly correlated, but the 22 kHz data is missing from figure 4D. I understand that this was likely done to keep the figure simple, but as only two frequencies show significant associations, I feel that it is important. Finally, the authors show that IHC synapse count is associated with ABR P1 amplitude at 16 kHz and 22 kHz only, not at the other four analyzed frequency regions. This is in contrast to some publications, that show a significant association between synapse loss and P1 amplitude across all frequencies in aged CBA/CaJ mice (See Sergeyenko et al., 2013), which were not discussed in this context.

Response: We apologize for the inappropriate presentation of the data and fully agree with the reviewer’s comment. Figures 4D and 4E have been revised accordingly. We also thank the reviewer for highlighting the inconsistency with the previous report. In our study, simple linear regression analysis of ABR P1 amplitude and synapse count showed statistically significant correlations only at 16 and 22 kHz, while other frequencies demonstrated a trend toward proportional relationships (p = 0.09 at 8 kHz; p = 0.08 at 32 kHz). We therefore speculate that the absence of intermediate-aged cohorts and the limited sample size may have reduced the power to detect significant correlations. This point has been added to the Discussion section (P.31, L.566–P.32, L.570).

3. Why were correlation analysis not performed for other data sets? For example, were there any associations between orphan ribbons, IHC count, or OHC count?

Response: We thank the reviewer for the constructive suggestion and have conducted correlation analyses for additional metrics. Significant correlations were also detected between IHC/OHC counts and ABR P1 amplitude (revised Fig. 3). A review of the literature indicates that IHC-specific loss leads to a reduction in the compound action potential^1^, while OHC loss and functional deficits are associated with reduced ABR P1 amplitude^2,3^. Based on these findings, we have considered the combined cochlear histological damage across these components as a contributing factor to the low ABR P1 amplitude observed in advanced-aged mice. This description and the corresponding citations have been added to the Discussion section (P.32, L.578–585).

*Reference #1: Qiu C, Salvi R, Ding D, Burkard R. Inner hair cell loss leads to enhanced response amplitudes in auditory cortex of unanesthetized chinchillas: evidence for increased system gain. Hear Res. 2000; 139(1-2): 153-171. https://doi.org/10.1016/s0378-5955(99)00171-9. PMID: 10601720.*

*Reference #2: Jung J, Joo SY, Min H, Roh JW, Kim KA, Ma JH, et al. MYH1 deficiency disrupts outer hair cell electromotility, resulting in hearing loss. Exp Mol Med. 2024; 56(11): 2423-2435. https://doi.org/10.1038/s12276-024-01338-4. PMID: 39482536.*

*Reference #3: Ingersoll MA, Lutze RD, Pushpan CK, Kelmann RG, Liu H, May MT, et al. Dabrafenib protects from cisplatin-induced hearing loss in a clinically relevant mouse model. JCI Insight. 2023; 8(24). https://doi.org/10.1172/jci.insight.171140. PMID: 37934596.*

4. In this paper, the authors found a unique difference in synapse counts between regions with minimal loss at 8 kHz. This is an interesting finding, but I have to respectfully disagree with the author’s hypothesis that this occurs due to it being the boundary area between 5.6 and the rest of the cochlea as to me it does not make logical sense. Yes, the ambient sound measured in this study was shifted towards lower frequencies, but the spectrum shows a relatively high level of energy between the 10^3 and 10^4, where 8 kHz is. Furthermore, as the sound presented in Feng et al., is white noise and theoretically should have equal energy spread across the entire spectrum, including the lower frequencies. In fact, Feng et al. found significant IHC synapse loss throughout the cochlea, from 10%-90%, even if it the loss was greatest at the 30-70% regions. That is to say, there is no ‘boundary’ in either the frequency spectrum recorded by the authors or in the citation used to justify this hypothesis.

Response: We thank the reviewer for the insightful comment and agree that our speculation regarding 8 kHz as a potential boundary area where synapses are less vulnerable to acoustic stimuli was not sufficiently supported. Upon careful review of our results and the relevant literature, we found it challenging to establish a reasonable hypothesis confirming that 8 kHz synapses are least susceptible to impairment from previously reported pathologies. We therefore suspect that unknown pathophysiological mechanisms, potentially influenced by combined factors such as individual variability, strain, sex, or rearing conditions (aside from ambient sound), may underlie this finding. Accordingly, we have eliminated the “boundary” hypothesis from the manuscript and revised the relevant sentences in the Discussion section (P.36, L.657–P.37, L.665).

Minor

- How many animals had thresholds at 85 dB SPL and how did this exclusion criteria affect the sample sizes for other audiometric measurements?

Response: We apologize for the ambiguity in the manuscript. In the aged group, we observed ABR thresholds of 85 dB SPL in four mice (eight ears) at 5.6 kHz, one mouse (two ears) at 8.0 kHz, one mouse (one ear) at 11.33 kHz, and one mouse (two ears) at 22.65 kHz. However, the exclusion criteria were applied only to ABR P1 metrics and not to threshold analysis. Therefore, the results already presented were not affected. To clarify this point, we have revised the Methods section accordingly (P.8, L.137–139).
