# Supplementary material for: Histopathological change of age-related hearing loss in female advance-aged CBA/CaJ mice
Source: PLoS One. 2025 Oct 7;20(10):e0334021. doi: 10.1371/journal.pone.0334021 (PMC12503338; doi:10.1371/journal.pone.0334021)
Supplement: Attachment — Submitted filename: Rev3_Response to Reviewers.docx [file pone.0334021.s017.docx]

**Point to Point Response to the Reviewers**

Manuscript Number: PONE-D-25-21282R3

We would like to thank the editor and the reviewers for their comments. We have re-edited and improved our manuscript as suggested. In the following, we provide a point-to-point response to every comment raised. We have highlighted portions of the manuscript that have been updated in response to the reviewers’ comments.

Reviewer #3:
All of my major comments have previous rounds have been addressed. However, there is a major issue in this submission. The authors state that they used the ABR Peak Analysis Software from Mass Eye and Ear Peabody Laboratories Engineering Core. They state that this software can automatically calculate, or extrapolate, thresholds lower than 20 dB SPL which was the lowest sound presentation level tested. This was strange to me, as I did not know of any software that could do this. I reached out to the team members of the Engineering Core who state that this piece of software cannot return a threshold lower than any of the tested levels. There is an option to auto-threshold, but if it detects that the threshold is lower than the lowest level tested, it warns the user and does not assign a threshold. Therefore, it seems impossible that the authors used this software to collect the audiometric thresholds reported in Figure 2 which are lower than 20 dB SPL. Was there a different software or recording method used to derive these thresholds other than those reported in the methods?

Although not a major component of the paper, this is a major concern for me as, unless there is software, analysis, or other recordings not reported in the methods, this is misinterpreted data at minimum.

Response: We thank the reviewer for this insightful comment and fully agree that an accurate description of the methods is essential to avoid misinterpretation of data. In our study, we analyzed the data using the ABR Peak Analysis Software downloaded from the Eaton-Peabody Laboratories Engineering Core, applied to recordings obtained with the Cochlear Function Test Suite in our facility. We recognize, however, that extrapolating thresholds without a detectable ABR waveform may underestimate the true threshold. To address this, we redefined ABR thresholds as 20 dB SPL only when clear waveforms were observed at a 20 dB SPL stimulus. Accordingly, we have reanalyzed the data and revised the Methods (P.7, L.128) and Results (P.16, L.297–P.17, L.300) sections, along with the relevant figures, table, and Supporting Information. Importantly, these adjustments did not affect our main conclusions.

I have a few other minor comments:

1. Please include non-significant statistics, especially if they are mentioned in the discussion. For example, when comparing the correlation between P1 amplitude and synapse count, only the statistics for the significant frequencies are reported. Then in the discussion, it's stated that other frequencies exhibit a trend towards a significant correlation, but the statistics for these correlations (including the p values) are not stated.

Response: We apologize for the insufficient data presentation that limited the connection between the Results and Discussion. We agree with the reviewer’s comment and have now added the *p* values across all frequencies for the correlation analysis between P1 amplitude and synapse count in the Results section (P.20, L.353–355).

2. Please cite Sergeyenko et al., 2013 on line 567 so that readers understand what previous report is being referred to.

Response: We thank the reviewer for the constructive comment. We have added the citation in the sentence as the reviewer’s suggestion. (P.32, L.568)
