# Supplementary material for: Histopathological change of age-related hearing loss in female advance-aged CBA/CaJ mice
Source: PLoS One. 2025 Oct 7;20(10):e0334021. doi: 10.1371/journal.pone.0334021 (PMC12503338; doi:10.1371/journal.pone.0334021)
Supplement: Attachment — Submitted filename: Rev4_Response to Reviewers.docx [file pone.0334021.s018.docx]

**Point to Point Response to the Reviewers**

Manuscript Number: PONE-D-25-21282R4

We would like to thank the editor and the reviewer for their comments. We have re-edited and improved our manuscript as suggested. In the following, we provide a point-to-point response to every comment raised. We have highlighted portions of the manuscript that have been updated in response to the reviewers’ comments.

Editor: Specifically: Please include the 20 dB SPL lower threshold limit in the limitations section of the discussion.

Reviewer #3: This is not contingent to acceptance, but I recommend that the authors include their 20 dB SPL lower threshold limit in the limitations section of the discussion.

Response: We thank the editor and reviewer for this valuable comment. In response, we have added the following sentence to the limitations section:

“A limitation of this study is that the lowest ABR threshold was set at 20 dB SPL, which may have restricted the evaluation of potential lower thresholds, particularly in the younger group.” (P.40, L.729-P.41, L.731)
